# Long-Term Vitamin D_3_ Supplementation Does Not Prevent Colonic Inflammation or Modulate Bone Health in IL-10 Knockout Mice at Young Adulthood

**DOI:** 10.3390/nu6093847

**Published:** 2014-09-22

**Authors:** Andrea J. Glenn, Kristina A. Fielding, Jianmin Chen, Elena M. Comelli, Wendy E. Ward

**Affiliations:** 1 Faculty of Medicine, University of Toronto, Toronto, ON M5S 3E2, Canada; E-Mails: andrea.glenn@utoronto.ca (A.J.G.); kristina.fielding@utoronto.ca (K.A.F.); jimmy.chen@utoronto.ca (J.C.); elena.comelli@utoronto.ca (E.M.C.); 2 Faculty of Applied Health Sciences, Brock University, St. Catharines, ON L2S 3A1, Canada

**Keywords:** vitamin D, intestinal inflammation, bone mineral density, bone strength, mice

## Abstract

Inflammatory bowel disease (IBD) is an idiopathic disease that can impair bone metabolism. Low vitamin D status has been implicated in its progress. This study used interleukin (IL)-10 knockout (KO) mice, that develop an intestinal inflammation when housed in a non-sterile environment, to determine if supplementation with vitamin D_3_ throughout life could mitigate inflammation and attenuate the lower bone mineral content (BMC) and density (BMD), and bone strength. Female IL-10 KO mice were randomized 25 or 5000 IU vitamin D_3_/kg diet throughout pregnancy and lactation. At weaning, offspring received the same or opposite diet as their mother until age three months. Body weight growth was similar among groups within a sex. At three months of age, there were no differences in inflammation and gene expression in the colon of offspring. Male offspring exposed to continuous 25 IU vitamin D_3_/kg diet had lower (*p* < 0.001) colonic VDR expression and those exposed only to low vitamin D_3 _ until weaning had higher serum IL-6. There were no differences in femur or vertebral BMC, BMD or bone strength. In summary, long-term exposure to vitamin D_3_ did not attenuate intestinal inflammation or preserve bone mineral or bone strength. Thus, supplementation with vitamin D_3_ does not exert anti-inflammatory effects in this mouse model that mimics human inflammatory bowel disease.

## 1. Introduction

Inflammatory bowel disease (IBD) is an idiopathic disease comprising Crohn’s disease and ulcerative colitis, characterized by a dysregulated immune response towards the normal intestinal microbiota in genetically susceptible people [1]. This results in the production of proinflammatory cytokines, such as interferon-γ (IFN-γ), interleukin-1α (IL-1α), interleukin-1β (IL-1β), interleukin-6 (IL-6), interleukin-17 (IL-17), and tumor necrosis factor-α (TNF-α) [2]. Of these, IL-1, IL-6, and TNF-α stimulate osteoclastogenesis, resulting in impaired bone metabolism [3]. Lower bone mineral density (BMD) and higher risk of fracture are indeed common in IBD patients, even among those not receiving treatment with glucocorticoids [4,5]. Thus, the inflammatory process itself causes deterioration of bone health, and bone health may be further compromised with glucocorticoid treatment. The prevalence of osteoporosis ranges from 12% to 42% among patients with IBD [4] and thus is a significant additional health concern. There are several different rodent models that are useful for studying the etiology of IBD and the potential benefits of an intervention. The interleukin-10 knockout (IL-10 KO) mouse model is one example that has proven useful in determining pharmacological and therapeutic treatments for IBD [6]. The IL-10 KO mouse spontaneously develops intestinal inflammation at six to eight weeks of age [7] and we have found that both male and female IL-10 KO mice fed control diet have impaired bone development by three months of age [8]. Specifically, BMC (bone mineral content) and BMD of the lumbar spine and peak load of an individual lumbar vertebra of both males and females were significantly lower than wild type controls.

The importance of vitamin D for bone health is well established in the literature. Thus, lower vitamin D status in IBD may contribute to poor bone health directly and may also lead to a dysregulated immune response and subsequent bone abnormalities in IBD [9,10]. Within the immune system, 1,25-dihydroxyvitamin D_3_ (1,25(OH)D_3_) inhibits T helper-1 type immune function in cells, induces T regulatory cells, and enhances phagocytosis by white blood cells [11]. 1,25(OH)D_3_ targets more than 900 genes [12], some of which are involved in intestinal homeostasis [13], which may play a role in IBD development [9,14]. In humans, early life nutrition [15] and season of birth [16] have been linked to later risk of developing IBD, indicating that a critical window for nutritional interventions to lower the risk of developing IBD exists. Similarly, vitamin D status has been shown to influence intestinal inflammation in mouse models. For example, vitamin D deficiency in weanling mice exacerbated the development of colitis after administration of dextran sodium sulfate [17]. Moreover, mice that cannot respond to vitamin D (vitamin D receptor (VDR) KO) or synthesize 1,25(OH)D_3_ (CYP KO) are particularly susceptible to the effects of dextran-sodium sulfate, experiencing more severe intestinal inflammation [18]. Supplementation with 1,25(OH)D_3_ prior to induction of colitis using dextran-sodium sulfate provided some protection against intestinal inflammation—suggesting a protective effect of 1,25(OH)D_3_ [18].

Vitamin D_3_ deficiency or an absence of VDRs have been found to exacerbate intestinal inflammation in IL-10 KO mice, resulting in premature death compared to IL-10 KO mice with adequate vitamin D status [19]. Treatment with 1,25(OH)D_3_ mitigated histologically-assessed inflammation [20] and down-regulated several TNF-α-associated genes in the colon of these mice [19]. However, intervention with vitamin D_3_ rather than 1,25(OH)D_3_, has not been studied in the IL-10 KO mouse. The objective of this study was to determine if lifelong supplementation with vitamin D_3_ *in utero* and through to three months of age could mitigate the development of intestinal inflammation and subsequent bone abnormalities (lower BMC, BMD and bone strength) in IL-10 KO offspring at young adulthood.

## 2. Experimental Section

### 2.1. Animals and Diet

All experimental procedures followed the policies set out by the Canadian Council on Animal Care [21] and were approved by the Animal Ethics Committee at the University of Toronto. Six breeding pairs of 129/SvEvIL-10 KO mice were a gift from Dr. Richard Fedorak, University of Alberta. Upon arrival, the breeding pairs were caged together in standard environmental conditions (12 h light:12 h dark cycle, room temperature of 23 °C) and fed AIN93G diet (Dyets Inc., Bethlehem, PA, USA). Incandescent lighting was used in the room to ensure that it was free of UVB radiation. Female offspring were weaned at postnatal day (PND) 29 and randomized to 1 of 2 modified AIN93G diets with (1) 5000 IU vitamin D_3_/kg diet (high (H), Diet# 119290) or (2) 25 IU vitamin D_3_/kg diet (low (L), Diet# 119289) (Table 1). Both diets contained 5 g of calcium/kg diet. At seven weeks of age, 28 female offspring were mated harem style and kept on their respective diet throughout pregnancy and lactation (H or L). Male and female offspring were weaned at PND 29 and randomized to either continue on the same diet as their mother or the other diet (H or L) from weaning through to the end of the study (three months of age). This resulted in four vitamin D_3_ interventions: HH, HL, LH, or LL where the first letter represents the diet consumed by dams during pregnancy and lactation and the second letter represents the diet consumed by offspring from weaning through to three months of age. Since this was a first study using the IL-10 KO mouse to study the effect of a dietary vitamin D_3_ intervention, a high and low level of vitamin D_3_ was specifically chosen to generate serum 25(OH)D levels of (≥50 nmol/L) and (<30 nmol/L), respectively, to elucidate potential biological responses [22]. Dams and offspring were euthanized at weaning and at three months of age, respectively. At necropsy, blood was drawn by cardiac puncture, a 1 cm portion of the proximal colon was cut and fixed in 10% phosphate-buffered formalin and the remainder was cut in half, snap frozen in liquid nitrogen and stored at −80 °C. Femurs and vertebrae were excised, cleaned of soft tissue, wrapped in saline soaked gauzed and stored at −80 °C.

### 2.2. Litter Characteristics and Body Weight

Pups were counted within the first week of life to determine litter size and sexed by measuring anogenital distance. Pup weight was measured at PND 14 during the suckling period. Body weight of offspring was measured at one and three months of age.

**Table 1:** Composition of modified AIN93G purified rodent diet containing high or low levels of vitamin D.

Ingredient	Unit	Value
***Macronutrients***		
Crude Protein	%	17.9
Crude Fat	%	7.0
Crude Fibre	%	4.8
Moisture	%	7.0
Ash	%	4.2
***Amino Acids***		
Arginine	%	0.70
Lysine	%	1.48
Methionine	%	0.56
Cystine	%	0.30
Tryptophan	%	0.21
Histidine	%	0.51
Leucine	%	1.76
Isoleucine	%	1.14
Phenylalanine	%	0.96
Tyrosine	%	0.98
Threonine	%	0.76
Valine	%	1.00
***Minerals***		
Calcium	mg/kg	5000
Phosphorus	mg/kg	1561
Potassium	mg/kg	3600
Sodium	mg/kg	1019
Magnesium	mg/kg	507
Iron	mg/kg	35
Zinc	mg/kg	30
Manganese	mg/kg	10
Copper	mg/kg	6.0
Iodine	mg/kg	0.2
***Vitamins***		
Vitamin A	IU/g	4.00
Vitamin D3	IU/g	0.025 ^†^ or 5.00 ^††^
Alpha-Tocopherol	IU/g	75.00
Thiamine	mg	5.0
Riboflavin	mg	6.0
Niacin	mg	30
Pantothenic Acid	mg	15.0
Pyridoxine	mg	6.0
Folic Acid	mg	2.0
Biotin	mcg	200
Vitamin B12	mcg	25.0
Vitamin K	mcg	750
**Gross Energy**	**kcal/g**	**3.80**

^†^: Vitamin D content of modified low vitamin D AIN93G diet (Diet #119289); ^††^: Vitamin D content of modified high vitamin D AIN93G diet (Diet #119290).

### 2.3. Serum 25(OH)D

Serum 25(OH)D levels were measured using the automated IDS-iSYS 25OHD chemiluminescence immunoassay (Immunodiagnostic Systems Inc., Fountain Hills, AZ, USA), following the manufacturer’s instructions.

### 2.4. Histological Assessment

Ten samples per group for mothers and 13 samples per group per sex for pups were embedded in paraffin and sectioned in 5 μm sections. Sections were stained with hematoxylin and eosin (H & E) for light microscopy (400×). Slides were analyzed by an individual who was blinded to the intervention (A.G), and the degree of inflammation was assessed on a scale of 0–4 as previously described and validated [8,23,24]. A minimum of three sections (one transverse, two longitudinal) and five fields per section (15 scores in total) were examined to determine the mean histological inflammatory score for each mouse.

### 2.5. RNA Extraction

Total RNA was extracted from 1 half of the proximal colon of 6 male mice at 3 months of age per group using *mir*Vana™ miRNA Isolation Kit (Ambion, Carlsbad, CA, USA), following the manufacturer’s protocol and stored at −80 °C. The concentration and purity of RNA samples were assessed using a Nanodrop 1000 Spectrophotometer (Nanodrop Technologies, Wilmington, DE, USA) (OD_260/280_, OD_260/230_). RNA quality was further confirmed by an Agilent 2100 Bioanalyzer (Agilent, Santa Clara, CA, USA).

### 2.6. Microarray Analysis

RNA samples used for microarray had a RNA Integrity Number (RIN) of 6.5 or higher. 200 ng of the RNA samples were labeled using Illumina TotalPrep-96 RNA Amplification kit (Illumina Inc., San Diego, CA, USA) and 1.5 ng of the generated cRNA were hybridized onto the Mouse WG-6 V2 Bead chips (Illumina Inc., San Diego, CA, USA). Microarrays were hybridized, washed and scanned according to the manufacturer’s protocols. Microarray data was checked for overall quality using the LUMI package in Bioconductor and R (version 2.14.1) [25]. For data analysis, raw expression data was imported into Genespring v11.5.1 (Agilent Technologies, Santa Clara, CA, USA) and normalized using a quantile normalization function along with a “per probe” median centered normalization. All data analyses were performed on log2 transformed data. There were a total of 45,281 probes on the Illumina Mouse Whole Genome (Version 2, release 6) array (Illumina Inc., San Diego, CA, USA). Comparisons between groups were considered separately using false discovery rate corrected *T*-tests (Benjamini and Hochberg FDR *q* < 0.05) or regular *T*-tests (*p* < 0.05) if the former produced no significant results. In order to remove probes showing no expression overall, a filtering was applied such that only probes that were in the upper 80th percentile of the distribution of intensities in 80% of either of the groups under statistical comparison were allowed to pass. Unsupervised hierarchical clustering was performed using a Pearson centered distance metric under average linkage rules. Microarray data have been deposited at Gene Expression Omnibus (GEO) with the accession number (GSE61111) [26]. Microarray experiments were performed at the Princess Margaret Genomics Centre, Toronto, Canada.

### 2.7. Colonic VDR Expression

To quantify colonic VDR expression, 5 μm sections of the proximal colon of the male mice were treated with aqueous 3% H_2_O_2_ for 10 min. The VDR antigen was retrieved in heated Tris-EDTA buffer. The sections were incubated with rabbit anti-mouse VDR polyclonal antibody (Abcam, Cambridge, MA, USA) diluted at 1:3000 in diluent buffer (DakoCytomation, Dako Canada, Inc., Burlington, ON, Canada) overnight at 4 °C. The sections were then incubated with biotinylated goat anti-rabbit IgG (Santa Cruz Biotechnology, Santa Cruz, CA, USA) overnight at 4 °C. Streptavidin-horseradish peroxidase and AEC substrate (DakoCytomation, Dako Canada, Inc., Burlington, ON, Canada) were used to show the antigens. Slides were read blindly (J.C.) with a light microscope (400×) and a total of 15 fields per mouse were assessed using the Allred scoring method [23].

### 2.8. Cytokine and Bone Marker Analysis

Serum levels of IL-1α, IL-6, IL-17, TNF-α, RANKL, and OPG were quantified using MILLIPLEX^®^ MAP Mouse Cytokine/Chemokine Magnetic Bead Panel Kit or MILLIPLEX^®^ MAP Mouse RANKL and OPG kits (Millipore Corporation, Billerica, MA, USA).

### 2.9. Bone Morphometry, Bone Mineral Content (BMC), BMD and Biomechanical Strength Testing

BMC and BMD of whole femurs, the proximal 1/3 of the femur and lumbar vertebra 4 were determined using pDEXA^®^ SABRE^™^ dual-energy x-ray bone densitometry (Orthometrix, White Plains, NY, USA) and a specialized software program (Host Software Version 3.9.4; Scanner Software version 1.2.0). All bones were scanned in air with a scan speed of 2 mm/min and at a resolution of 0.01 × 0.01 mm as previously described [27]. BMC represents the total mineral content of the measured bone (whole femurs, lumbar vertebra 4) or region of a bone (1/3 proximal femur) while BMD represents the amount of mineral per area measured. Three-point bending (femurs), femur neck fracture and compression (LV4) were performed using a Materials Testing System (Model 4442, Instron Corp., Norwood, MA, USA) fitted with customized jigs and data were analyzed using specialized software (Bluehill 2) [27]. All tests were performed with a crosshead speed of 2 mm/min until fracture occurred.

### 2.10. Statistical Analyses

Statistical analyses were performed using Sigma Stat (Version 3.5, Jandel Scientific, Chicago, IL, USA). Results are expressed as mean ± SEM and statistical significance was set at *p* < 0.05. Litter characteristics and dam data were analyzed by Student’s *t*-test. Body weight at weaning (1 month of age) and immediately prior to necropsy (three months of age) was analyzed by one-way ANOVA within sex at each time point. All other data was analyzed by two-way ANOVA, within sex, with mother’s diet and pup’s diet as main effects. Male and female data were analyzed separately because of known differences in growth and bone development. Bonferroni’s *post hoc* test was used when there was a significant main effect.

## 3. Results

### 3.1. Litter Characteristics and Body Weight

There were no significant differences in litter size (H: 6 ± 0 pups, L: 6 ± 0 pups, *p* = 0.51) or pup weight at PND 14 (H: 6.9 ± 0.3 g, L: 6.7 ± 0.2 g, *p* = 0.43) between high and low vitamin D groups. There were no differences in body weight growth among groups for either sex at one month of age (weaning) or three months of age (necropsy) (Table 2).

**Table 2:** Body weight of offspring at 1 month of age (weaning) and 3 months of age (necropsy).

Age	HH	HL	LH	LL	*p Value*
**Females**					
1 month (g)	12.1 ± 0.1	12.3 ± 1.1	12.4 ± 0.8	12.7 ± 0.6	0.96
3 months (g)	20.0 ± 1.0	20.3 ± 1.1	19.8 ± 1.2	20.2 ± 1.3	0.95
**Males**					
1 month (g)	13.7 ± 0.8	12.9 ± 1.1	14.5 ± 0.8	14.1 ± 0.0	0.10
3 months (g)	23.5 ± 0.7	23.7 ± 1.1	23.8 ± 1.8	23.5 ± 0.8	0.94

Data are means ± SEM, *n* = 16/group.

### 3.2. Serum 25(OH)D

For both male and female offspring, serum 25(OH)D levels of the HH and LH groups were higher (*p* < 0.001) than the HL and LL groups (Table 3).

### 3.3. Histological Assessment

**Figure 1:**
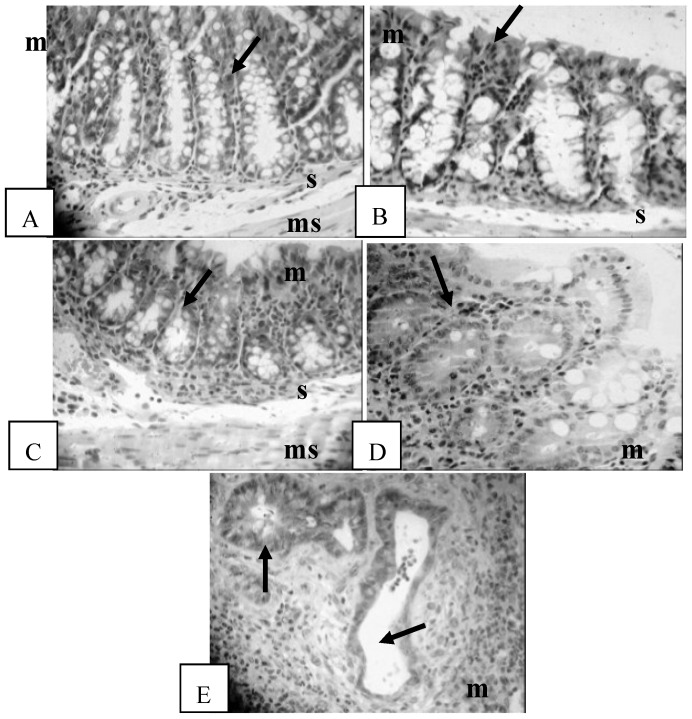
Histological assessment of colonic inflammation. (**A**) Score 0: no inflammation with normal crypt and goblet cells (arrow); m: mucosa; s: submucosa; ms: muscularis. H & E, 400×; (**B**) Score 1: minimal evidence of inflammatory infiltrate (arrow), H & E, 400×; (**C**) Score 2: significant evidence of inflammatory infiltrate (arrow), H & E, 400×; (**D**) Score 3: significant evidence of inflammatory infiltrate with goblet cell depletion (arrow), H & E, 400×; (**E**) Score 4: severe inflammation characterized by widespread infiltration with inflammatory cells, formation of crypt abscesses (arrows), mucosal thickening, submucosal cell infiltration, a decrease in goblet cells and destruction of architecture, H & E, 400×.

Evidence of inflammation such as lymphocyte infiltration, goblet cell depletion, cryptitis, crypt abscesses and complete destruction of architecture was observed in the mice studied. Examples of each inflammatory score from the IL-10 KO mice in this study are shown in Figure 1. There were no significant differences in the inflammation severity score in the proximal colon among the four vitamin D interventions for either the male and female mice (Table 3). There were no significant differences in the inflammation severity scores between dams in the high and low (H: 2.1 ± 0.2, L: 2.3 ± 0.2, *p* = 0.10) vitamin D groups. The inflammation severity score of the dams did not correlate with the inflammation severity score of offspring (data not shown).

**Table 3:** Colon severity score, serum 25(OH)D and cytokines of offspring at three months of age.

Outcomes	HH	HL	LH	LL	*Mother’s Diet*	*Pup’s Diet*	*Interaction*
**Females**							
Colon severity score	1.4 ± 0.1	1.1 ± 0.1	1.1 ± 0.3	1.4 ± 0.3	0.57	0.56	0.07
Serum 25(OH)D (nmol/L)	99.1 ± 4.1 ^a^	22.7 ± 1.5 ^b^	93.6 ± 7.5 ^a^	20.6 ± 0.6 ^b^	0.39	<0.001	0.70
IL-1α (pg/mL)	153.5 ± 47.9	77.8 ± 55.3	175.2 ± 47.9	125.9 ± 60.6	0.51	0.25	0.81
IL-6 (pg/mL)	7.6 ± 7.6	12.9 ± 9.8	17.8 ± 7.6	28.8 ± 7.6	0.13	0.33	0.73
IL-17 (pg/mL)	17.0 ± 4.8	8.9 ± 4.8	14.8 ± 5.4	14.2 ± 4.8	0.75	0.39	0.45
TNF-α (pg/mL)	10.7 ± 2.6	6.1 ± 2.6	12.8 ± 2.8	7.4 ± 2.6	0.52	0.07	0.88
**Males**							
Colon severity score	1.5 ± 0.2	1.5 ± 0.2	1.4 ± 0.2	1.7 ± 0.2	0.93	0.26	0.84
Serum 25(OH)D (nmol/L)	93.8 ± 2.7 ^a^	26.0 ± 1.6 ^b^	101.6 ± 10.3 ^a^	23.0 ± 2.2 ^b^	0.66	<0.001	0.34
IL-1α (pg/mL)	60.6 ± 13.9	62.9 ± 18.1	42.2 ± 18.1	42.2 ± 18.0	0.28	0.94	0.96
IL-6 (pg/mL)	15.6 ± 7.1	13.9 ± 7.6	30.7 ± 7.1	37.3 ± 8.7	0.01	0.75	0.60
IL-17 (pg/mL)	26.4 ± 4.9	21.9 ± 5.2	20.8 ± 4.9	25.3 ± 5.6	0.83	0.99	0.39
TNF-α (pg/mL)	12.3 ± 2.1	10.9 ± 2.3	10.2 ± 1.9	11.3 ± 2.3	0.68	0.94	0.57

Data are means ± SEM. Means in a row with superscripts without a common letter differ; *p* ≤ 0.05; Colon severity score (*n* = 13/group); serum 25(OH)D_3_ (*n* = 5/group); cytokines (*n* = 9–10/group).

### 3.4. Colonic Gene Expression

A total of 31,439 probes were considered to be expressed for the post-weaning diet group comparisons (HH, LH *versus* HL, LL) and 31,447 probes were considered to be expressed for the pre-weaning diet group comparisons (HH, HL *versus* LH, LL), based on the criteria explained in the Experimental Section. There were no differentially expressed genes among the four vitamin D interventions and there was no clustering of samples based on their gene expression profiles (Figure S1).

### 3.5. Colonic VDR Expression

**Figure 2:**
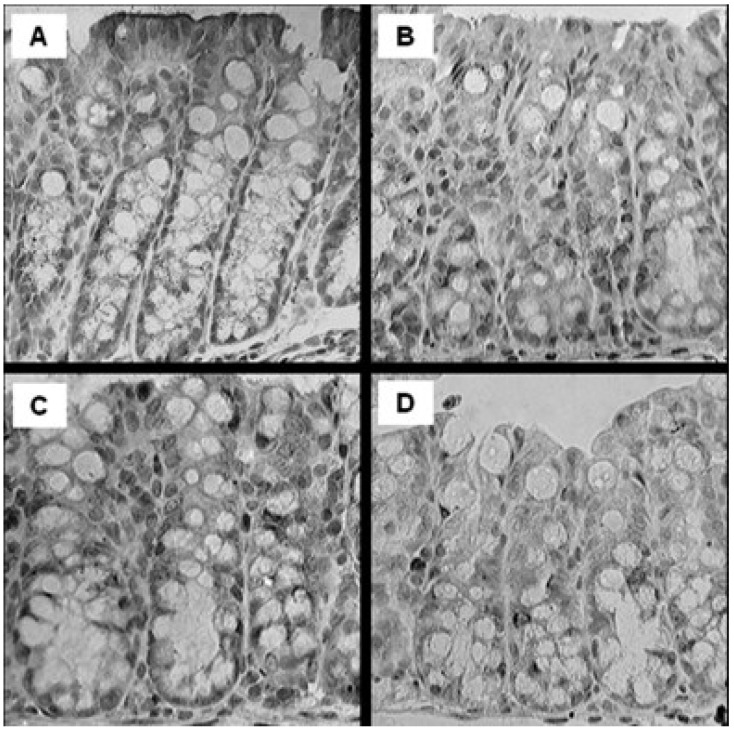
Representative microphotography of proximal colonic VDR expression in male offspring IL-10 KO mice fed diet supplemented with high vitamin D or low vitamin D. (A) Group HH; (B) Group HL; (C) Group LH; (D) Group LL. 400×. Showing mucosa layer only. *n* = 13/group.

In male but not female offspring, the immunoactivity of VDR staining (Figure 2) in the HH (score 4.1 ± 0.2), HL (score 3.6 ± 0.2) and LH (score 3.9 ± 0.2) groups were strongest, with weaker immunoactivity (*p* < 0.001) in the LL (2.7 ± 0.3) group compared to all other interventions.

### 3.6. Cytokine Analysis

There were no significant differences in serum IL-1α, IL-6, IL-17 and TNF-α in male and female offspring (Table 3). However, male mice exposed to low vitamin D_3_ pre-weaning had higher levels of IL-6 than male mice exposed to high vitamin D_3_ in early life (*p* = 0.01).

### 3.7. Serum Bone Markers and Femur and Lumbar Vertebrae Outcomes

There were no significant differences in serum RANKL and OPG concentrations among male mice (Table 4). Among females, RANKL was significantly higher (*p* < 0.01) in the LL group compared to the HL group with no significant differences in OPG (Table 4). There were no differences in BMC or BMD of the whole femur, 1/3 proximal femur and lumbar vertebrae 4 in either sex (Table 4). Similarly, peak load at the femur midpoint, femur neck and lumbar vertebrae did not differ among groups for either sex (Table 4). Dams who consumed a high vitamin D_3_ diet (H: 20.6 ± 0.5 N) for three weeks prior to breeding and throughout pregnancy and lactation had a higher (*p* = 0.006) peak load at the femur midpoint compared to those who consumed a low vitamin D_3_ diet (L: 18.3 ± 0.6 N). Femur neck peak load (H: 9.2 ± 0.5 N, L: 10.4 ± 0.4 N, *p* = 0.10) and LV2 (H: 47.3 ± 1.7 N, L: 44.6 ± 1.2 N, *p* = 0.21) of dams did not differ between groups.

**Table 4:** Bone outcomes of offspring at three months of age: serum markers, biomechanical bone strength, and bone mineral content and density.

Bone Outcomes	HH	HL	LH	LL	*Mother’s Diet*	*Pup’s Diet*	*Interaction*
**Females**							
RANKL (pg/mL)	115 ± 10 ^a,b^	87 ± 10 ^a^	115 ± 9 ^a,b^	139 ± 10 ^b^	0.009	0.85	0.01
OPG (pg/mL)	1795 ± 182	1829 ± 182	1501 ± 172	1681 ± 193	0.23	0.56	0.68
OPG/RANKL ratio	16 ± 2	25 ± 5	12 ± 2	13 ± 2	0.004	0.06	0.16
Femur midpoint yield load (N)	10.1 ± 0.2	8.7 ± 0.4	9.4 ± 0.3	9.1 ± 0.4	0.66	0.005	0.11
Femur midpoint peak load (N)	19.8 ± 0.6	18.4 ± 0.4	18.6 ± 0.6	18.2 ± 0.4	0.17	0.09	0.34
Femur neck peak load (N)	10.0 ± 0.4	9.6 ± 0.4	10.1 ± 0.4	9.8 ± 0.3	0.71	0.33	0.84
LV_2_ peak load (N)	45.4 ± 1.9	44.1 ± 1.5	47.3 ± 1.5	46.2 ± 1.4	0.21	0.45	0.95
Whole femur BMC (mg)	20.7 ± 0.74	19.8 ± 0.89	19.1 ± 0.73	19.1 ± 0.94	0.20	0.92	0.34
Whole femur BMD (mg/mm^2)^	6.49 ± 0.16	6.41 ± 0.19	6.23 ± 0.18	6.18 ± 0.18	0.15	0.67	0.92
1/3 prox. femur BMC (mg)	7.92 ± 0.24	7.62 ± 0.33	7.27 ± 0.25	7.52 ± 0.32	0.20	0.92	0.34
1/3 prox. femur BMD (mg/mm^2^)	6.95 ± 0.15	6.87 ± 0.18	6.72 ± 0.19	6.62 ± 0.16	0.17	0.62	0.96
LV_4_ BMC (mg)	6.20 ± 0.39	6.49 ± 0.21	5.95 ± 0.38	5.63 ± 0.35	0.10	0.96	0.36
LV_4_ BMD (mg/mm^2^)	5.48 ± 0.01	5.73 ± 0.16	5.25 ± 0.29	5.29 ± 0.26	0.26	0.62	0.72
**Males**							
RANKL (pg/mL)	116 ± 10	119 ± 10	119 ± 10	109 ± 10	0.74	0.72	0.49
OPG (pg/mL)	2016 ± 214	1992 ± 226	2027 ± 240	2154 ± 226	0.70	0.82	0.74
OPG/RANKL ratio	19 ± 2	18 ± 4	20 ± 5	22 ± 4	0.52	0.89	0.77
Femur midpoint yield load (N)	10.5 ± 0.26	9.95 ± 0.28	1.50 ± 0.02	10.36 ± 0.36	0.22	0.11	0.91
Femur midpoint peak load (N)	21.84 ± 0.60	20.45 ± 0.50	10.83 ± 0.35	21.41 ± 0.76	0.91	0.43	0.18
Femur neck peak load (N)	9.82 ± 0.38	9.30 ± 0.43	10.82 ± 0.54	10.08 ± 0.58	0.07	0.20	0.82
LV_2_ peak load (N)	51.6 ± 1.3	47.4 ± 1.3	51.7 ± 1.5	49.9 ± 2.2	0.43	0.07	0.44
Whole femur BMC (mg)	20.4 ± 0.49	19.8 ± 1.02	21.8 ± 0.76	19.5 ± 0.66	0.56	0.07	0.22
Whole femur BMD (mg/mm^2^)	6.18 ± 0.12	6.11 ± 0.18	6.46 ± 0.17	5.96 ± 0.12	0.65	0.07	0.16
1/3 prox. femur BMC (mg)	8.00 ± 0.22	7.71 ± 0.37	8.38 ± 0.31	7.64 ± 0.34	0.61	0.10	0.48
1/3 prox. femur BMD (mg/mm^2^)	6.63 ± 0.13	6.53 ± 0.19	6.88 ± 0.18	6.47 ± 0.13	0.54	0.12	0.33
LV_4_ BMC (mg)	6.30 ± 0.27	5.60 ± 0.21	6.39 ± 0.39	5.98 ± 0.25	0.17	0.55	0.56
LV_4_ BMD (mg/mm^2^)	5.12 ± 0.24	5.04 ± 0.18	5.29 ± 0.28	4.91 ± 0.10	0.93	0.27	0.46

Data are means ± SEM. Means in a row with superscripts without a common letter differ; *p* ≤ 0.05. Serum bone markers (*n* = 9–10/group); bone strength outcomes (*n* = 14–16/group), BMC and BMD (*n* = 10/group).

## 4. Discussion

Our study demonstrated that high dietary vitamin D_3_ does not mitigate intestinal inflammation or concomitant bone abnormalities in the IL-10 KO mouse model. Since this was a first study using the IL-10 KO mouse to study the effect of a dietary vitamin D_3_ intervention, a low and a high level of dietary vitamin D_3_ was specifically chosen to elucidate potential biological responses [22]. Thus, even the high level of dietary vitamin D_3_—that resulted in markedly elevated serum 25(OH)D levels—was not sufficient to regulate colonic gene expression. Since serum concentrations of 25(OH)D_3_ are a strong predictor of serum 1,25(OH)D_3_ concentrations [28], serum 1,25(OH)D_3_ would be expected to be markedly higher with the high vitamin D diet. The circulating level of 1,25(OH)D_3_ from our study is estimated to be >200 pmol/L based on previous study by others that fed mice the same level of vitamin D used in the present study [22]. Because serum 1,25(OH)D_3_ was not directly measured, it is possible that the conversion to 1,25(OH)D_3_ is compromised in the IL-10 KO model and may thereby explain the lack of effect. Moreover, while we specifically measured inflammation in the colon, a highly inflamed site in this mouse model and also an intestinal region that is exposed to a significant antigenic load, the small intestine (jejunum) may have been more responsive to the intervention [7]. Others studying the effects of 1,25(OH)D_3_, in IL-10 KO mice have shown jejunum to be responsive without reporting effects in colon [20]. Of note is that the diet was supplemented with calcium, double the level of calcium provided in AIN93G and in the present study, as the authors noted that 1,25(OH)D_3_ attenuates autoimmune encephalomyelitis to a greater extent in the presence of supplemental calcium [20]. Intervention with 1,25(OH)D_3_ in combination with supplemental calcium has also been shown to downregulate production of TNF-α in the colon [19]. These findings suggest that the active form of vitamin D_3_ as well as supplemental calcium may be needed to elicit an effect in this mouse model.

The gene expression findings in this study were unexpected because there was significantly lower protein level of VDR in the proximal colon of LL offspring. The lower colonic VDR expression suggests that lifelong exposure to low vitamin D may be detrimental to intestinal health. VDR KO mice have impaired immunity and are more susceptible to IBD when given a chemical insult [29]. Furthermore, IL-10/VDR double KO mice have a more severe form of disease [19]. Therefore, if the mice in this study were administered a chemical insult or pathogen, the LL group may be more susceptible to these insults. Interestingly, a study investigating the effects of the VSL#3 probiotic mixture on gene expression in IL-10 KO mice discovered differentially expressed genes and a reduced histopathology score in the caecum, but not in the colon, between the control and probiotic treated mice [30]. The researchers explained that the finding of no effect in the colon may be due to the differences in microbial retention and colonization between the gut segments studied, or that there was a lower level of inflammation in the caecum. Perhaps this is also why there were no differences in gene expression or histopathology scores in the colon in our study.

While bone abnormalities have been previously reported in both male and female IL-10 KO mice compared to wild type at three months of age [8,31], there were only modest differences in bone outcomes among IL-10 KO groups receiving different levels of vitamin D_3_. These findings suggest that the normal level of calcium in both diets (5000 mg/kg diet) may compensate for the lower vitamin D in the diet. The dietary calcium requirements for mice are not particularly evidence-based with respect to bone health [32] and were largely based on preventing kidney calcification that was common with the previous diet (AIN76). In developing the current AIN93G diet, Reeves *et al.* [32] compared the current level of 5000 mg/kg diet (or 0.5%) to a higher (0.67% calcium) and lower level of calcium (0.33%) while maintaining a calcium to phosphorus molar ratio of 1.3 to prevent kidney calcification. The sole outcome of bone health was tibia content of calcium, phosphorus and magnesium content. The level of calcium in tibia was similar, despite the range of intakes (0.33%–0.67% calcium) and supports the fact that calcium may be present in excess of need for bone development. Our previous study showed that skeletal sites that differ in the amount of trabecular or cortical bone such as femur midpoint and lumbar vertebra, are compromised in IL-10 KO mice compared to wild type mice, but only vertebra have both a lower quantity of bone mineral and reduced strength [8]. In the present study, in which mice were the same age as those in the previous study that showed that bone abnormalities are present at three months of age [8], the only benefit to bone was observed in female offspring consuming a high vitamin D_3_ diet post-weaning. These females had a significantly higher yield load at the femur midpoint—a site mainly composed of cortical bone—compared to those consuming a low vitamin D diet. That there was no benefit to lumbar vertebra, a site that tends to be more responsive to dietary interventions, is interesting and may be due to the fact that the mice are undergoing overall rapid body growth, as well as cortical bone growth. The fact that most bone outcomes do not differ despite the markedly lower serum 25(OH)D_3_ emphasizes the need to better understand requirements of vitamin D in mice and the potential influence of altering or not altering the level of calcium in a widely-used control diet (AIN93G). Dams supplemented with high vitamin D_3_ had higher peak load at the femur midpoint compared to the low vitamin D group_3_, suggesting that the vitamin D intervention was of sufficient duration to modulate bone health.

Choice of model system for studying intestinal inflammation and bone health modulates the effect of a dietary vitamin D_3_ intervention. For example, others have used an adoptive IL-10^−/−^ CD4+ T cell transfer model of chronic colitis in which piroxicam is administered to induce acute colitis [33]. In this study, a high (5000 IU/kg diet) or low (1000 IU/kg diet) dietary vitamin D_3_ was administered for 12 days after a seven-day intervention with piroxicam and showed that high vitamin D_3_ exacerbated the effect of colitis on bone metabolism [33]. Of note is that administering piroxicam resulted in a more severe phenotype than in our study as body weight loss was approximately 20% after administration. We observed no loss in body weight throughout the study.

The higher level of serum RANKL in the LL group of females compared to the HL group, indicates a potential protective effect of bone health with a diet high in vitamin D_3_ during early life. It is not clear why this relationship was not observed among males. RANKL is a member of the TNF cytokine family and functions as a key factor for osteoclast differentiation and activation, indicating a higher level may be detrimental to bone health [34]. Furthermore, there was an effect of mother’s diet on the ratio of OPG to RANKL in female offspring. Females exposed to high vitamin D_3_ pre-weaning had a higher ratio compared to those exposed to low vitamin D_3_ pre-weaning. This effect did not persist at three months of age but suggests that early-life exposure to supplemental vitamin D_3_ may be beneficial for bone health. Patients with juvenile osteoarthritis with bone erosions have been found to have a lower ratio of OPG to RANKL than patients with juvenile osteoarthritis without bone erosions, indicating a lower ratio is suggestive of bone damage [34].

## 5. Conclusions

In conclusion, supplementation with vitamin D_3_ beginning early in life did not attenuate intestinal inflammation while low vitamin D_3_ throughout life resulted in lower colonic VDR expression in the colon. Moreover, there were no effects on BMC and BMD and only modest effects on bone strength. Supplementation with vitamin D_3_ rather than pharmacological 1,25(OH) D_3_ may not be potent enough to exert anti-inflammatory effects in this model of IBD.

## Supplementary Files

Supplementary File 1
