# Supplementary material for: Long-Term Vitamin D3 Supplementation Does Not Prevent Colonic Inflammation or Modulate Bone Health in IL-10 Knockout Mice at Young Adulthood
Source: Nutrients. 2014 Sep 22;6(9):3847–62. doi: 10.3390/nu6093847 (PMC4179191; doi:10.3390/nu6093847)

**Supplementary Information**

**Figure S1.** This dendogram shows the hierarchical clustering (unsupervised; Pearson centered correlation with average linkage rules) of proximal colon transcriptomes obtained from microarray analysis of 24 samples (*n* = 6 males/group). Numbers indicate each male mouse within each group.


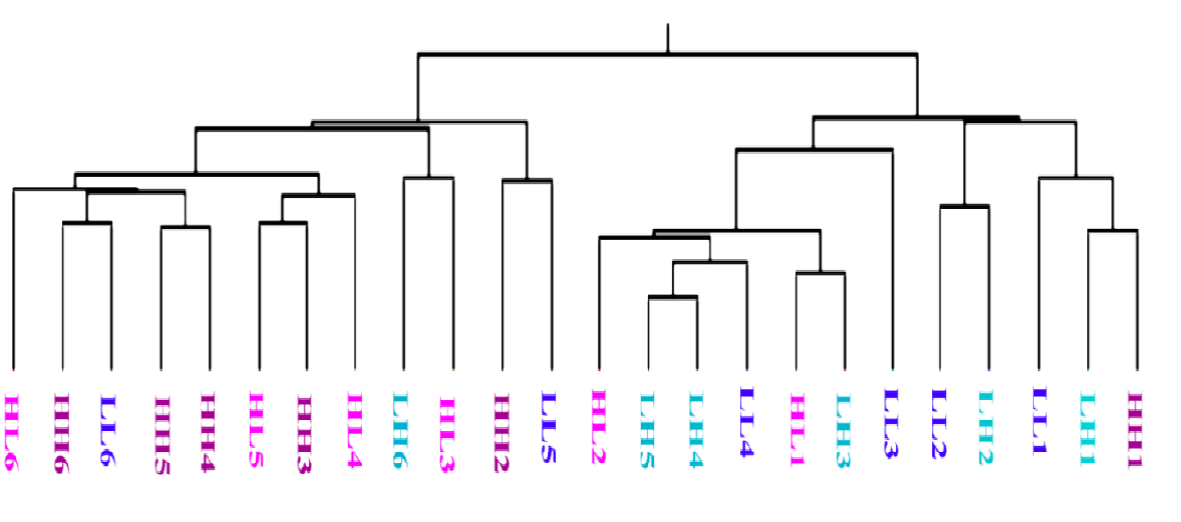

Supplement: Supplementary File 1 [file nutrients-06-03847-s001.docx]
